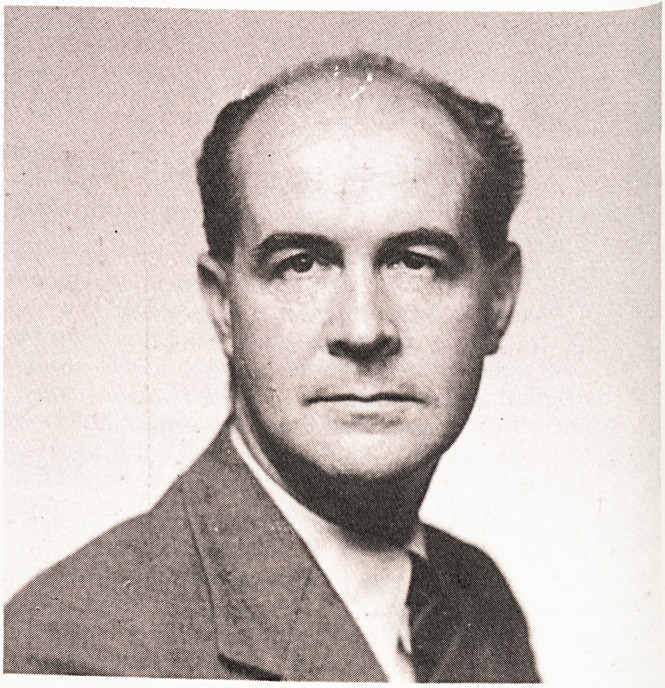# Dr James Macrae

**Published:** 1987-08

**Authors:** A. T. M. Roberts


### James Macrae TD, MD, FRFRSG, FRCP (Glas), DPH.

Dr James Macrae, who was Physician Superintendant of Ham Green Hospital, Bristol from 1948 to 1976 died on 5th February 1987 aged 75 years.

He was born on 24th February 1911 at Kyle of Lochalsh and educated at Dingwall Academy and Glasgow University where he qualified in 1934. During the following 5 years he held junior hospital posts in virtually every medical and surgical speciality and in 1939 was appointed Deputy Medical Superintendant of Lanarkshire County Hospital in Motherwell. In February 1940 he joined the Royal Army Medical Corps but shortly before leaving for the Middle East he married Phyl whom he had met in Guernsey in 1939 during his first real holiday since qualification. He served continuously for 4½ years in Palestine, the Western Desert, Italy and Jugoslavia before returning to the UK in 1945 with the rank of Lieut. Colonel. Subsequently he served in the Territorial Army for a further 20 years.

After demobilisation he became deputy Medical Superintendant in Weston super Mare and in 1948 was appointed Consultant General Physician to Ham Green Hospital and worked there continuously until his retirement in 1976.

With the wide experience he had had before, during and after the war Jimmy was a classic example of the devoted whole time Medical Superintendant who could turn his skills to practically every branch of clinical medicine and surgery. His appointment to Ham Green coincided with the effective chemotherapy for most of the major infectious diseases and he had the thrill and pleasure of taking a major part in the virtual eradication of many of these previously dreaded conditions. But other challenges arose and Jimmy tackled them with skill and ingenuity; not only was he a wise physician but he had a remarkable mechanical aptitude.

In the 1950s with the upsurge of poliomyelitis he pioneered the development of assisted positive pressure respiration and built most of the early apparatus himself with the help of a local garage engineer. In 1959 he published in the Lancet the second account of such treatment in the UK—the first having appeared in the same journal only the week previously from Oxford. He personally performed nearly 250 tracheotomies for diphtheria, poliomyelitis and other forms of respiratory failure and in 1962 he carried out the first renal dialysis to be performed in the Bristol Clinical Area on a patient with renal failure due to drinking antifreeze. The first patient to be accepted for maintenance dialysis is still alive and in 1968 Humphrey White performed the first renal transplant in Bristol on one of his patients.

During his years at Ham Green Jimmy was virtually never off duty by day or by night and was an inspiration to his junior staff for his dedication and the standard of medicine which he set. He was greatly admired by all for his modesty and his devotion to his patients and his opinion was eagerly sought by consultants and general practitioners alike. For 17 years he was treasurer of the Bristol Medico-Chirurgical Society and his Presidential Address in 1972 was on "Edward Jenner—a great Englishman". It was very appropriate that he was a founder member of the Jenner trust set up in Bristol in 1967.

In 1968 Jimmy had a stroke from which he made a reasonable recovery but in 1982 his wife Phyl also suffered a stroke, thereafter they lived quietly in retirement until his peaceful end. He is survived by Phyl and their daughter Judy.

A. T. M. Roberts.

## Figures and Tables

**Figure f1:**